# Natural CD4^+^ T-Cell Responses against Indoleamine 2,3-Dioxygenase

**DOI:** 10.1371/journal.pone.0034568

**Published:** 2012-04-23

**Authors:** Shamaila Munir, Stine Kiaer Larsen, Trine Zeeberg Iversen, Marco Donia, Tobias Wirenfeldt Klausen, Inge Marie Svane, Per thor Straten, Mads Hald Andersen

**Affiliations:** 1 Department of Hematology and Oncology, Center for Cancer Immune Therapy (CCIT), Copenhagen University Hospital, Herlev, Herlev, Denmark; 2 Department of Biomedical Sciences, University of Catania, Catania, Italy; University of Montreal, Canada

## Abstract

**Background:**

The enzyme indoleamine 2,3-dioxygenase (IDO) contributes to immune tolerance in a variety of settings. In cancer IDO is expressed within the tumor itself as well as in antigen-presenting cells in tumor-draining lymph nodes, where it endorses the establishment of peripheral immune tolerance to tumor antigens. Recently, we described cytotoxic CD8^+^ T-cell reactivity towards IDO-derived peptides.

**Methods and Findings:**

In the present study, we show that CD4^+^ helper T cells additionally spontaneously recognize IDO. Hence, we scrutinized the vicinity of the previously described HLA-A*0201-restricted IDO-epitope for CD4^+^ T-cell epitopes. We demonstrated the presence of naturally occurring IDO-specific CD4^+^ T cells in cancer patients and to a lesser extent in healthy donors by cytokine release ELISPOT. IDO-reactive CD4^+^ T cells released IFN-γ, TNF-α, as well as IL-17. We confirm HLA class II-restriction by the addition of HLA class II specific blocking antibodies. In addition, we detected a trend between class I- and class II-restricted IDO responses and detected an association between IDO-specific CD4^+^ T cells and CD8^+^ CMV-responses. Finally, we could detect IL-10 releasing IDO-reactive CD4^+^ T cells.

**Conclusion:**

IDO is spontaneously recognized by HLA class II-restricted, CD4^+^ T cells in cancer patients and in healthy individuals. IDO-specific T cells may participate in immune-regulatory networks where the activation of pro-inflammatory IDO-specific CD4^+^ responses may well overcome or delay the immune suppressive actions of the IDO-protein, which are otherwise a consequence of the early expression of IDO in maturing antigen presenting cells. In contrast, IDO-specific regulatory T cells may enhance IDO-mediated immune suppression.

## Introduction

Indoleamine 2,3-dioxygenase (IDO) has attracted much interest, since it is involved in the generation of immune tolerance in a variety of physiological and pathological settings. The immunological effects of IDO are mainly limited to acquire peripheral tolerance or unresponsiveness to novel antigens. Thus, it does not seem to be required for constitutive tolerance to self antigens. Hence, systemic inactivation at the organism level, either pharmacologically or genetically, does not appear to cause severe autoimmunity [1]. IDO mediated degradation of the essential amino acid tryptophan to kynurenine and other downstream metabolites suppresses effector T-cell function [2], [3]. In addition, this seem to facilitate the conversion of naïve T lymphocytes into Tregs [4], [5]. IDO can be expressed by a variety of cell types, including dendritic cells (DC), tumor cells and stoma cells. In cancer, IDO is involved in the induction of tolerance towards tumor antigens and to facilitate immune escape [6], [7].

Consistent with a role for IDO in mediating tolerance to tumors, preclinical studies have shown the promise of IDO inhibitors in the targeting of several cancers [8]–[14]. We have recently described the presence of CD8^+^, cytotoxic IDO-reactive T cells in peripheral blood of both cancer patients and healthy donors. We demonstrated that IDO-specific CD8^+^ T cells were able to recognize and kill tumor cells including directly isolated AML blasts as well as IDO-expressing DC, i.e. one of the major immune suppressive cell populations [15]. Furthermore, we showed that the presence of such IDO-specific CD8^+^ T cells boosted T-cell immunity against viral or tumor-associated antigens by eliminating IDO^+^ suppressive cells [16]. Hence, IDO-specific effector T cells may play a vital role for the mounting or maintaining of an effective adaptive immune response.

In the present study, we show that IDO is in addition the target for CD4^+^ T-helper cells.

## Materials and Methods

### Donors

Peripheral Blood Mononuclear Cells (PBMC) were collected from healthy individuals and cancer patients (renal cell carcinoma, melanoma, and breast cancer). The PBMC from cancer patients were obtained prior to entering into clinical trials, which were concurrently approved by the Danish Medicines Agency and registered at www.clinicaltrials.gov. Identifier (renal cell carcinoma trial: NCT00197860, melanoma trials: NCT00978913 & NCT00197912, breast cancer trial: NCT00197925). Written informed consent from the donors was obtained before study entry. All patients had histological verified metastatic disease (stage IV TNM classification) at inclusion. Blood samples from cancer patients were drawn a minimum of four weeks after termination of any kind of anti-cancer therapy. The majority of renal cell carcinoma patients had previously been treated with IL-2 and IFN-α, most melanoma patients had received high dose IL-2 and IFN-α, while all breast cancer patients were pre-treated with several kinds of chemotherapy, (e.g. epirubicin, docetaxel, cabecitabine), trastuzumab, and/or endocrine therapy. PBMC were isolated using lymphoprep separation, HLA-typed (Department of Clinical Immunology, University Hospital, Copenhagen, Denmark) and frozen in FCS with 10% DMSO. The protocols were approved by the Scientific Ethics Committee for The Capital Region of Denmark and conducted in accordance with the provisions of the Declaration of Helsinki.

### Peptides

Three synthetic peptides were synthesized (TAG Copenhagen, Copenhagen, Denmark): DTLLKALLEIASCLE (IDO194-208) entitled IDO194, LLEIASCLEKALQVF (IDO200-214) entitled IDO200 as well as DTLLKALLEIASCLEKALQVF (IDO194-214) entitled IDOlong. Although the IDO1 and IDO2 proteins share 43% identity at the amino acid level, only 5 out of 21 amino acids from the IDOlong peptide sequence can be found identical in the IDO2 protein sequence. Additional peptides included: HLA-A2-restricted ALLEIASCL (IDO199-207) entitled IDO5; the HLA-A2 -restricted epitope CMV pp65_495–503_ (NLVPMVATV), the HLA-A11-rstricted epitope CMV pp65_16–24_ (GPISGHVLK), the HLA-A1 and HLA-A24 restricted epitope CMV pp65_341–350_ (QYDPVAALFF) or the HLA-A1 restricted epitope CMV pp65_363–373_ (YSEHPTFTSQY). Finally a nonsense peptide (GARVERVDFGNFVFNISVLW) was used as irrelevant control.

### ELISPOT assay

The ELISPOT assay was used to quantify peptide-specific IFN-γ, TNF-α or IL-17 releasing effector cells as described [17]. PBMC were stimulated once *in vitro* with peptide or with DC pulsed with peptide prior to analysis as described [18] to extend the sensitivity of the assay. After seven days in culture with 25 µg/ml peptide and 20 U/ml IL-2 (PeproTech, London, UK), cells were counted and analyzed in ELISPOT. Briefly, nitrocellulose bottomed 96-well plates (MultiScreen MAIP N45; Millipore) were coated overnight with IFN-γ, TNF-αor IL-17 capture mAb (Mabtech, Nacka Strand, Sweden). The wells were washed, blocked by X-vivo medium. PBMC or isolated CD4^+^ cells with or without DC were added in duplicates at different cell concentrations, with or without 5 µg/ml peptide. The plates were incubated overnight. The following day, medium was discarded and the wells were washed prior to addition of appropriate biotinylated secondary mAb (Mabtech). The plates were incubated at room temperature (RT) for 2 hours, washed, and Avidin-enzyme conjugate (AP-Avidin; Calbiochem/Invitrogen Life Technologies) was added to each well. Plates were incubated at RT for 1 hour and the enzyme substrate NBT/BCIP (Invitrogen Life Technologies) was added to each well and incubated at RT for 5–10 min. Upon the emergence of dark purple spots, the reaction was terminated by washing with tap water. The spots were counted using the ImmunoSpot Series 2.0 Analyzer (CTL Analyzers). CD4^+^ cells were isolated either using CD4 MicroBeads or EasySep human CD4^+^ cell enrichment kit according to manufacturers protocol by Miltenyi Biotech or Stem Cell Technology, respectively. The purity was subsequently analyzed by FACS on a FACS Canto using CD3-FITC, CD4-PE and CD8-APC conjugated antibodies (BD Biosciences). For class II-restriction assays 2 µg/ml HLA class II antibody (Monoclonal Antibody (MBS140186), MyBioSource, San Diego, US) were added to ELISPOT wells for ½ hour before the addition of IDOlong peptide.

For *ex vivo* ELISPOT analysis, CD4 T-cells were purified and directly plated on IFN-γ capture mAb coated plate with or without IDOlong-pulsed autologous DC. To correlate IDOlong responses with HLA class I-restricted IDO5 or CMV epitopes, *in vitro* or *ex vivo* IFN-γ ELISPOT was performed as described above. For the induction of IDO specific T-cells by Cytosine-phosphate-Guanine (CpG) ODN (Type B CpG oligodeeoxynucleotide specific for human TLR9; InvivoGen), PBMC were stimulated with 40 U/ml IL-2 with and without 1 ug/ml of CpG for two weeks before analysis. For the examination of patient characteristics and of the nature of cytokines concomitantly secreted by each patient, IDO responders were defined as average number of antigen specific cells ±½ standard deviation >25 per 5×10^5^ PBMC.

### Intracellular Staining for IFN-γ, TNF-α, Il-17

For detection of cell subpopulations producing cytokines, PBMC that were cultured for seven days in the presence of peptide as described for ELISPOT, were stimulated with IDOlong (5 µg/ml) for 5 hours at 37°C with 5% CO_2_ in air. Positive control wells were set-up with the addition of SEB (5 µg/ml). GolgiPlug (BD) was added at a dilution of 1∶200 after the first hour of incubation. After 4 additional hours cells were washed twice with PBS, stained fluorochrome conjugated antibodies for surface markers (CD3-Amcyan, CD4-PerCP and CD8-Pacific Blue, all from BD). Cells were washed one additional time and thereafter fixed and permeabilized with Fixation/Permeabilization and Permeabilization Buffer (eBioscience), according to manufacturer’s instructions. Cells were subsequently stained with fluorochrome-conjugated antibodies for intracellular cytokines. The following combinations were used: IFNγ-PeCy7 (BD), TNFαAPC (eBioscience) and IL-17a-FITC (eBioscience). Relevant isotype controls were used to enable correct compensation and confirm antibody specificity. At least 10^5^ CD4^+^ T cells were acquired. Stained cells were analyzed using a BD FACSCanto II flow cytometer. Analysis was performed with BD FacsDiva Software.

For the generation of IDOlong specific bulk cultures PBMC were stimulated twice with autologous DC matured with IDOlong peptide and 120 U/ml IL-2. Seven days after the second stimulation, PBMC were stimulated with autologous DC matured either with an irrelevant 20 amino acid long nonsense peptide, IDOlong peptide, lysate from the IDO^+^ colon cancer cell line SW480 (available at the American Type Culture Collection (ATCC)), or lysate from the IFN-γ pre-treated (100 U/ml for three days) melanoma cell line MM404.111 for 5 hours. After stimulation, intracellular cytokine staining was performed as described above in this section. Expression of IDO by SW480 and IFN-γ pre-treated MM404.111 was confirmed by RT-PCR (data not shown).

## Results

### Selection of a 21 Amino Acid Long Peptide from IDO

Previously, we characterized an HLA-A2-restricted, IDO-derived CD8^+^ T cell epitope from IDO199-207 (ALLEIASCL). This peptide is included in an ongoing first-in-man phase I vaccination trial at Center for Cancer Immune Therapy, Herlev Hospital, Denmark in patients with non small cell lung cancer (NSCLC) (www.clinicaltrials.gov; NCT01219348). In the present study, we sat out to examine if IDO in addition serve as target for CD4^+^ immune responses. Instead of examining spontaneous immune response against overlapping peptides covering all of the 403 amino acids in the IDO sequence, which would require large quantities of human PBMC, we focused on the region IDO199-207, which have previously been described to be immunogenic. In this respect, it has been described that T-cell epitopes often cluster together at specific immunogenic regions. Thus, we examined the region containing the HLA-A2-restricted epitope for potential class II epitopes using the predictive algorithm developed by Rammensee et al. available at www.syfpeithi.de.” [19]. The algorithm selected a 15 mer peptide DTLLKALLEIASCLE (IDO194-208) to be a high affinity HLA-DRB1*0101-restricted epitope and a 15 mer peptide LLEIASCLEKALQVF (IDO200-214) to be a high affinity HLA-DRB1*0301-restricted epitope. Consequently, we synthesized a 21 amino acid peptide entitled IDOlong containing both peptides (DTLLKALLEIASCLEKALQVF (IDO194-214)). The predictive algorithm additionally proposed that this sequence contains HLA-DRB1*0401, HLA-DRB1*0701 as well as HLA-DRB1*1501 15 mer epitopes.

### Spontaneous Cytokine Release in Response to IDOlong Peptide

**Figure 1:**
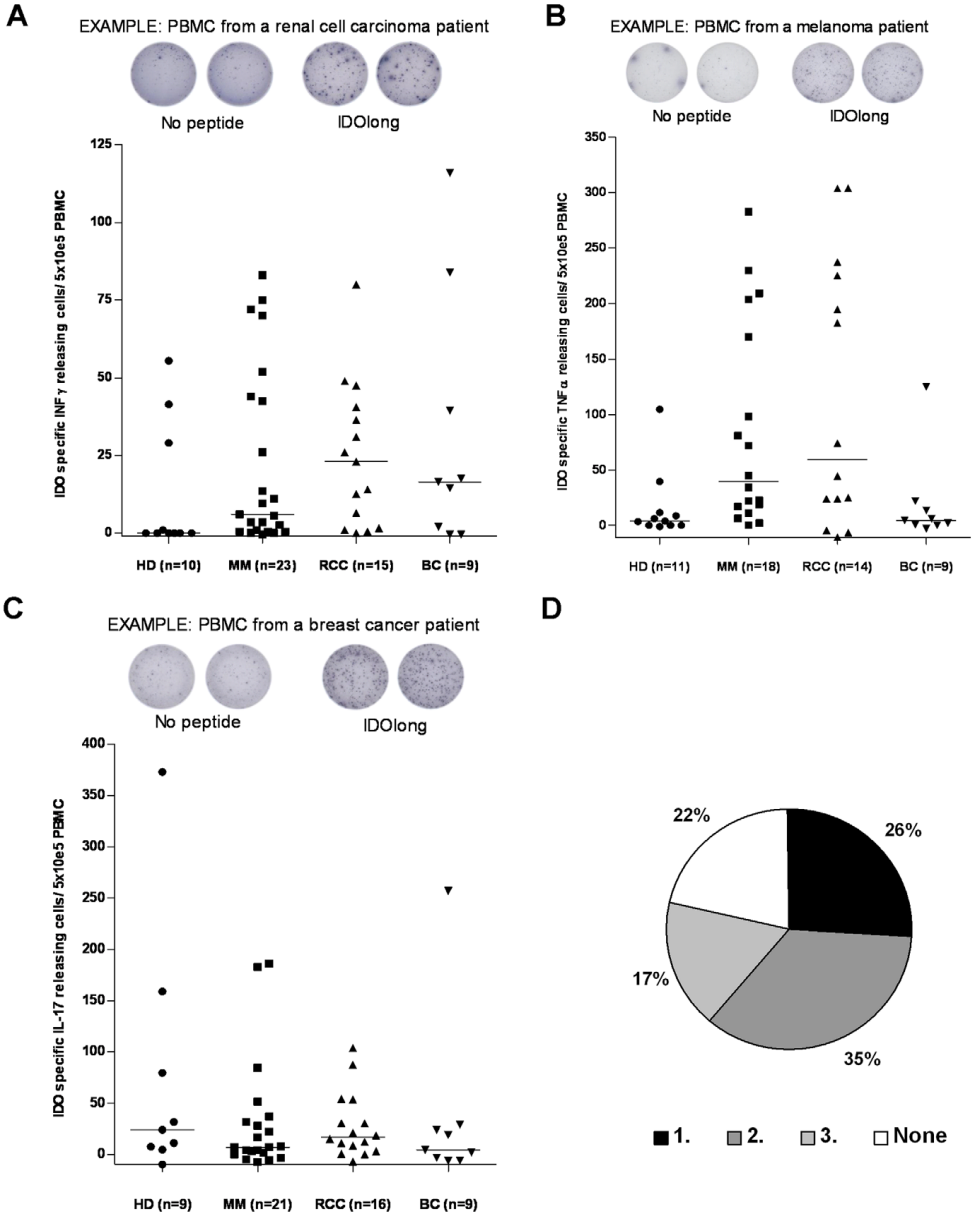
T-cell responses against IDOlong. *(* ***A*** *),* T-cell responses against IDOlong (DTLLKALLEIASCLEKALQVF (IDO194-214)) as measured by IFN-γELISPOT: Example of an ELISPOT response against IDOlong in PBMC from a renal cell carcinoma patient (*Top*). The average number of IDOlong-specific spots (after subtraction of spots without added peptide) was calculated per 5×10^5^ PBMC for each patient. PBMC from ten healthy individuals (HD), twenty three melanoma patients (MM), fifteen renal cell carcinoma patients (RCC) as well as nine breast cancer patients (BC) were analyzed. PBMC were stimulated once with peptide before being plated at 5×10^5^ cells per well in duplicates either without or with the IDOlong peptide (*bottom*). *(* ***B*** *),* T-cell responses against IDOlong as measured by TNF-α ELISPOT: Example of an ELISPOT response against IDOlong in PBMC from a melanoma patient (*Top*). The average number of IDOlong-specific spots (after subtraction of spots without added peptide) was calculated per 5×10^5^ PBMC for each patient. PBMC from eleven healthy individuals (HD), eighteen melanoma patients (MM), fourteen renal cell carcinoma patients (RCC) as well as nine breast cancer patients (BC) were analyzed. PBMC were stimulated once with peptide before being plated at 5×10^5^ cells per well in duplicates either without or with the IDOlong peptide (*bottom*). *(* ***C*** *),* T-cell responses against IDOlong as measured by IL-17 ELISPOT: Example of an ELISPOT response against IDOlong in PBMC from a breast cancer patient (*Top*). The average number of IDOlong-specific spots (after subtraction of spots without added peptide) was calculated per 5×10^5^ PBMC for each patient. PBMC from nine healthy individuals (HD), twenty one melanoma patients (MM), sixteen renal cell carcinoma patients (RCC) as well as nine breast cancer patients (BC) were analyzed. PBMC were stimulated once with peptide before being plated at 5×10^5^ cells per well in duplicates either without or with the IDOlong peptide (*bottom*). *(* ***D*** *),* Percentage of donors secreting none *(white)*, one *(black)*, two *(dark grey)* or all three *(light grey)* cytokines (IFN-γ,TNF-α,IL-17) in response to IDOlong.

**Figure 2:**
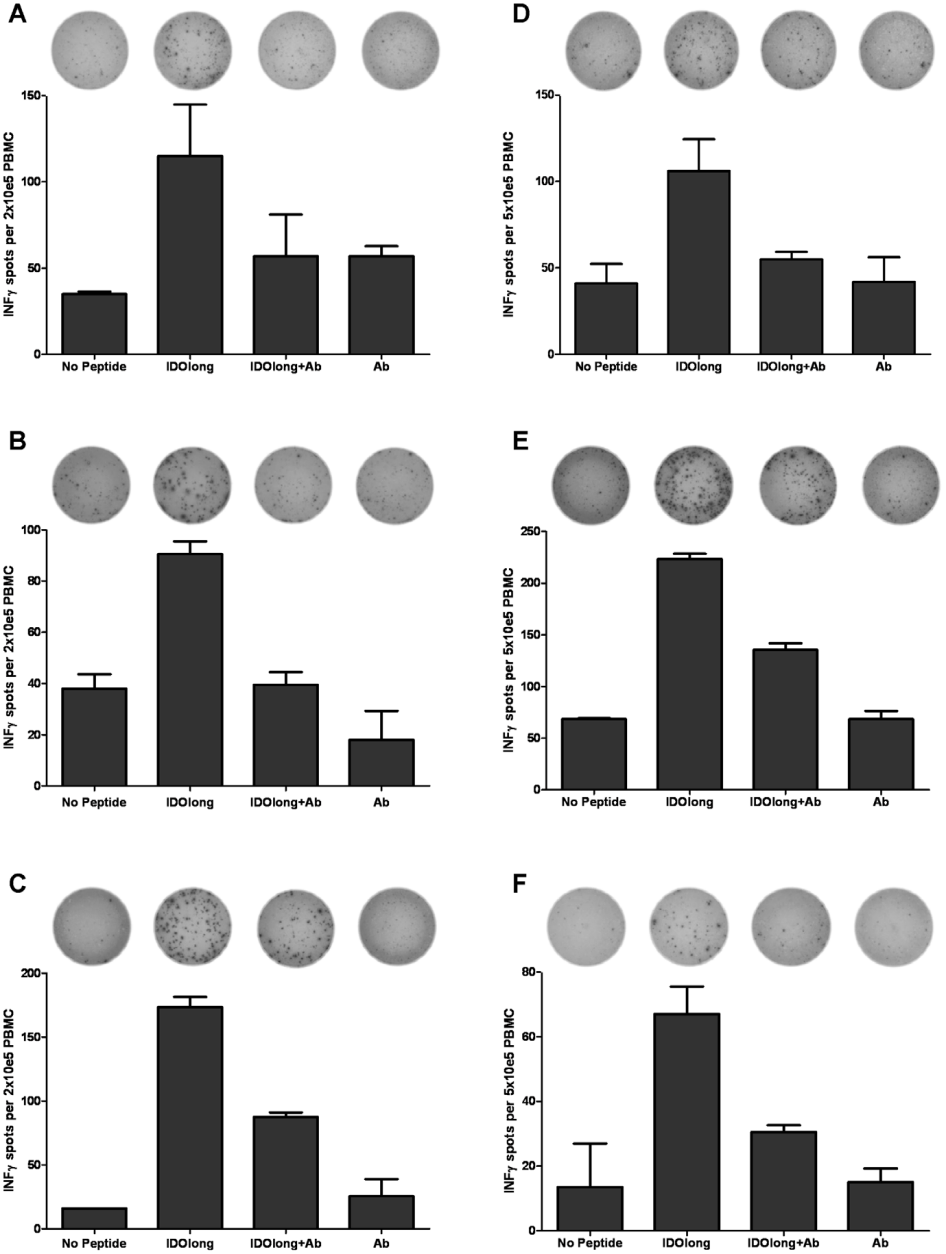
HLA class II-restricted IDOlong responses as measured by IFN-γELISPOT. PBMC from one renal cell carcinoma patient *(* ***A*** *)* and five melanoma patients *(* ***B,C,D,E,F*** *)* were stimulated once with peptide before being plated at either 2×10^5^ or 5×10^5^ cells per well in duplicates either without or with the IDOlong peptide or without and with a monoclonal anti-HLA class II antibody. The average number of IFN-γ releasing cells was calculated either per 2×10^5^ PBMC or 5×10^5^ PBMC for each patient.

**Figure 3:**
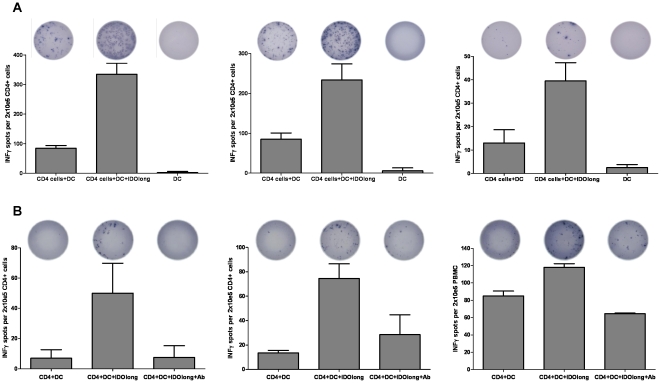
CD4^+^ T cell responses against IDO. *(* ***A*** *),* CD4^+^ T cell responses against IDOlong as examined by IFN-γ ELISPOT. PBMC from two malignant melanoma patients *(left, middle)* and one renal cell carcinoma patient *(right)* were stimulated once with IDOlong peptide before CD4^+^ T cells were isolated. The CD4^+^ cells were plated at 2×10^5^ cells per well in duplicates with 10^4^ DC either without or with the IDOlong peptide. As a control 10^4^ DC were plated alone without T cells. The numbers of IFN-γ releasing cells were counted for each patient. *(* ***B*** *), ex vivo* ELISPOT analysis of CD4 T-cells isolated from three melanoma patients. CD4^+^ cells were isolated from PBMC from three different donors and directly plated at 2×10^5^ cells per well in duplicates with 10^4^ DC either without or with the IDOlong peptide. HLA-class II antibody were added to two additional wells with CD4 cells, dendritic cells and IDOlong peptide. The numbers of IFN-γ releasing cells were counted for each patient.

Using the IFN-γ, TNF-α, IL-17 ELISPOT secretion assays, we examined peripheral blood T cells from cancer patients and healthy individuals for the presence of specific T-cell responses against IDOlong. Thus, PBMC from late stage cancer patients (breast cancer, melanoma and renal cell carcinoma) were stimulated once with the IDOlong peptide *in vitro* before examination by ELISPOT. Frequent ELISPOT responses against IDOlong were detected in IFN-γ, TNF-α as well as IL-17 assays (Figure 1, 2 and 3). Furthermore, we found that circulating IDO-specific, CD4^+^ T cells were present in healthy donors. We recently described that healthy individuals likewise harbor CD8^+^ specific T-cell responses [16]. We further assayed if we could detect responses against IDOlong directly in *ex vivo* ELISPOT in PBMC from eight patients. The frequency of IDO-reactive T cells were markedly increased by *in vitro* stimulation, although IDO-reactive T cells were readily detectable *ex vivo* in one breast cancer patient (150 IDOlong reactive cells/6×10^5^ PBMC; data not shown). Figure 1D illustrates that we could detect immune response against at least one cytokine in almost 80% of the examined donors. In 26% we detected a response against one of either IFN-γ, TNF-α or IL-17, in 35% against two cytokines and in 17% of the donors we detected a response against all three cytokines.

### Class II-restriction of IDOlong Responses

Next, we confirmed that the CD4 T-cell responses against IDOlong were indeed HLA class II-restricted responses. Hence, we examined the effect of the addition of anti-HLA class II blocking antibody to ELISPOT assays with PMBC from six IDOlong responding donors. As shown in figure 2 the IDOlong-responses were robustly inhibited by anti-HLA class II blocking antibodies in PMBC from all donors.

### CD4^+^ T Cell Responses Against IDOlong

We further confirmed that the ELISPOT responses indeed were CD4^+^ T-cell responses. Hence, CD4^+^ cells were isolated from three responding donors before examination by ELISPOT against DC without or pre-pulsed with IDOlong. The purity of the CD4 isolations was above 97% (data not shown). These measures confirmed that CD4^+^ T cells were indeed responsible for the response against the IDOlong peptide (Figure 3A). Next, we examined if IDOlong responses were detectable directly *ex vivo* without the need of a prior *in vitro* peptide stimulation step. Thus, CD4^+^ cells were isolated from PBMC from three different donors and directly added to ELISPOT assays with autologous DC with and without IDOlong. From figure 3 it can be seen that it was indeed possible to detect CD4 specific T-cell reactivity against IDO in the three donors. The IFN-γ release could be blocked by the addition of HLA-class II antibody (Figure 3B).

### Intracellular Cytokine Stainings

**Figure 4:**
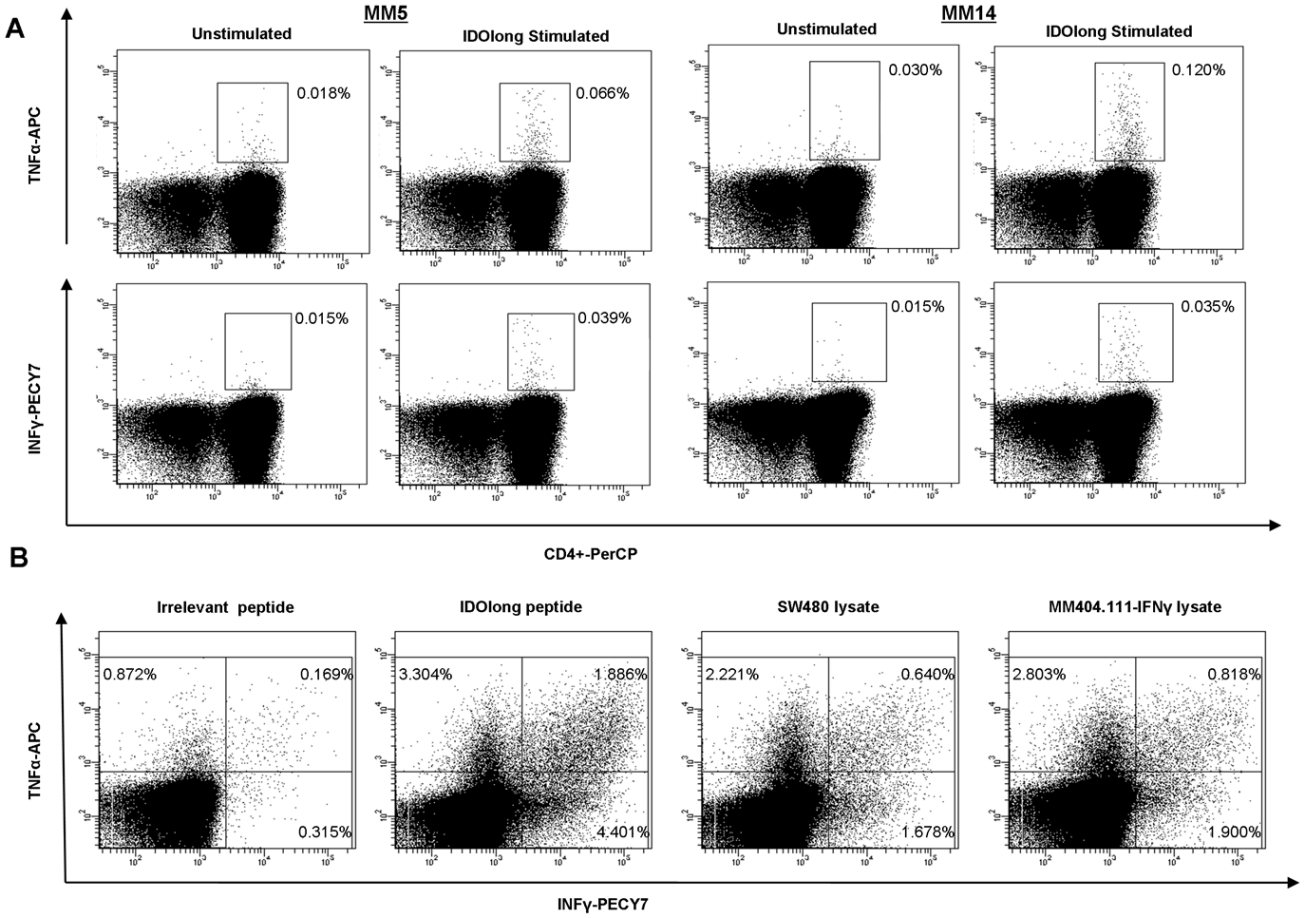
Intracellular cytokine staining. *(* ***A*** *),* TNF-α (*top*) and IFN-γ (*below*) intracellular cytokine stainings. PBMC from two melanoma patients were analyzed with and without IDOlong-stimulation. *(* ***B*** *),* PBMC from a melanoma patient were stimulated 2 times with autologous DC that had been matured with IDOlong peptide in the presence of IL-2 before being analysed by intracellular TNF-α and IFN-γ stainings. PBMC were stimulated with autologous DC matured with either an irrelevant 20 amino acid long nonsense peptide, IDOlong peptide, lysate from the IDO^+^ colon cancer cell line SW480 or lysate from the IFN-γ pre-treated melanoma cell line MM404.111. FACS plots were gated on live CD4^+^ T-cells.

The T-cell mediated recognition of IDOlong was additionally analyzed by intracellular cytokine stainings (ICS) (IFN-γ, TNF-α, IL-17) in six cancer patients. These analyses appeared less sensitive than the ELISPOT. Thus, we could not detect IL-17 release by ICS in any of the patients. However, in two melanoma patients we could detect a CD4^+^ specific release of TNF-α and to a lesser extend IFN-γ in response to IDOlong (figure 4A). Having identified donors hosting responses against the IDOlong peptide, we used PBMC from a responding melanoma patient to generate a bulk culture against this peptide *in vitro*. Subsequently, we *in vitro* stimulated PBMC with IDOlong-pulsed autologous DC. After three *in vitro* re-stimulations, the peptide specificity was tested using IFN-γ, TNF-α intracellular staining (figure 4B). This assay revealed that around 3% of the culture released IFN-γ in response to autologous DC pulsed with IDOlong, around 4.4% released TNF-α and almost 2% both cytokines. As a control we stimulated the bulk culture with autologous DC pulsed with a 20 amino acid long irrelevant, nonsense peptide (GARVERVDFGNFVFNISVLW) (figure 4B). Next, we examined the recognition of IDO^+^ tumor lysates by the IDOlong reactive bulk culture. Hence, we examined the bulk cultures reactivity against autologous DC pulsed with either lysate from the IDO^+^ colon cancer cell line SW480 or lysate from the melanoma cell line MM404.111. MM404.111 did not express IDO and was consequently pre-treated with IFN-γ. From figure 4B it can be seen that the bulk culture reacted towards both IDO^+^ tumor lysates.

### Correlation Between IDOlong Responses and CD8 T-cell Responses Against IDO as well as CMV

**Figure 5:**
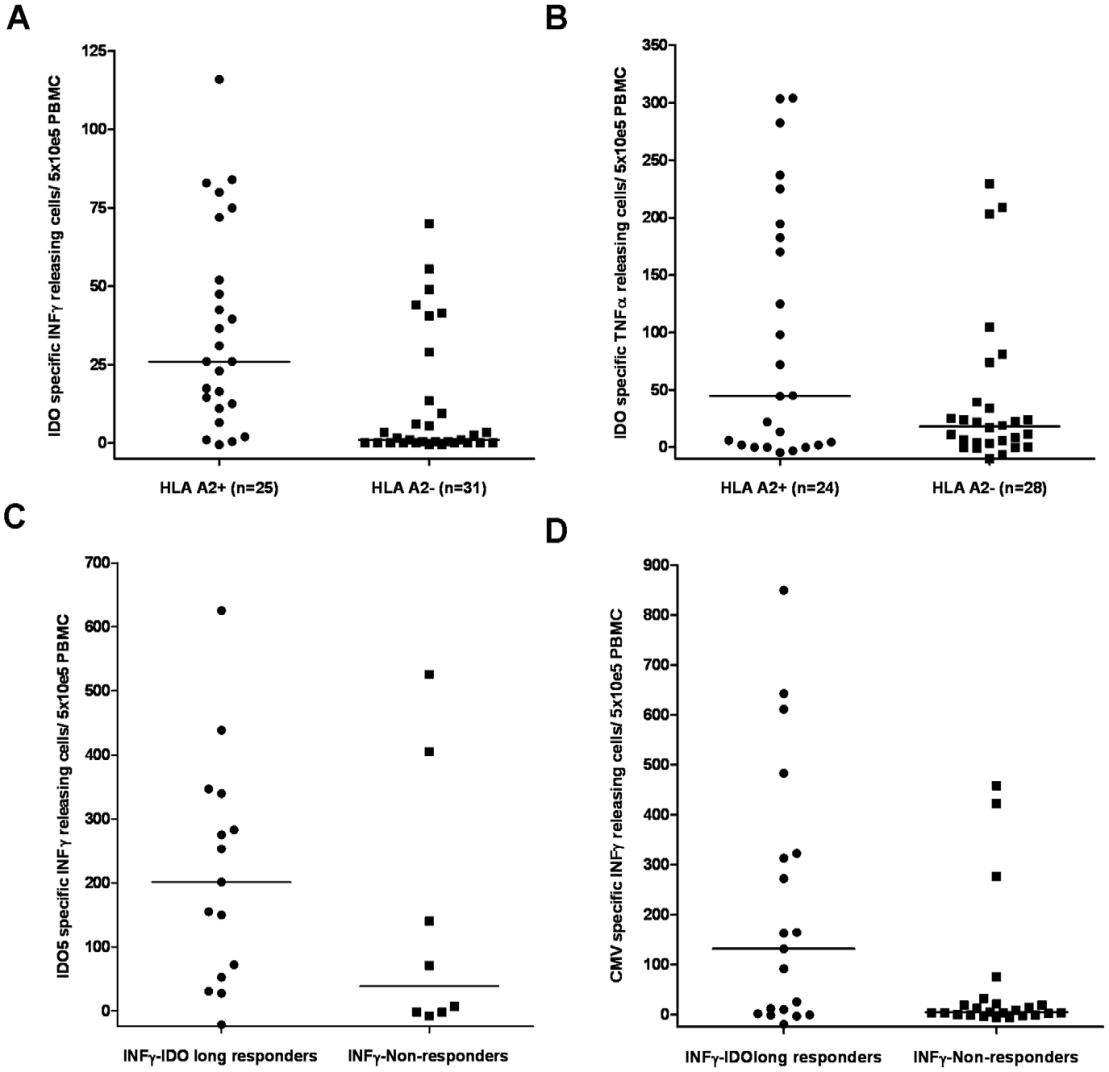
Correlation of CD4 responses against IDO with CD8 T-cell responses. *(* ***A*** *),* the correlation of IFN-γ IDOlong responses with the class I tissue type HLA-A2. A Mann-Whitney test demonstrated that for there was a significant difference between IFN-γ responders and non-responders (P = 0.0003). *(* ***B*** *),* the correlation of TNF-α IDOlong responses with the class I tissue type HLA-A2. A Mann-Whitney test demonstrated that there was not significant difference between TNF-α responders and non-responders (P = 0.2). *(* ***C*** *),* correlation between IFN-γ IDOlong responses and IFN-γresponses against the HLA-A2-restricted, IDO-derived epitope IDO5 (IDO199-207). PBMC from IDO responders and non-responders were stimulated with IDO5 once *in vitro* before being plated at 5×10^5^ cells per well in duplicates either without or with the IDO5 peptide. A Mann-Whitney test demonstrated that for there was not significant difference between responders and non-responders (P = 0.2). *(* ***D*** *),* correlation between IFN-γ IDOlong responses and IFN-γ responses against HLA class I-restricted epitopes from CMV. PBMC from IDO responders and non-responders were directly plated at 5×10^5^ cells per well in duplicates either without or with one of the most immunogenic HLA class I-restricted CMV-epitopes, i.e. the HLA-A2-restricted epitope CMV pp65_495–503_ (NLVPMVATV), the HLA-A11-restricted epitope CMV pp65_16–24_ (GPISGHVLK), the HLA-A1- and HLA-A24-restricted epitope CMV pp65_341–350_ (QYDPVAALFF) or the HLA-A1-restricted epitope CMV pp65_363–373_ (YSEHPTFTSQY) depending on the HLA-type of the individual donor. A Mann-Whitney test illustrated that there was significant difference in T cell reactivity towards CMV epitopes between IDOlong responders and non-responders (P = 0.04).

The IDOlong peptide contains the region IDO199-207, which cover the HLA-A2-restricted, IDO-derived CD8^+^ T cell epitope named IDO5. Thus, first we examined the correlation between IDOlong responses and the class I tissue type HLA-A2 (figure 5A and 5B). Indeed, more IDOlong responders were identified among HLA-A2 positive donors. A Mann-Whitney test demonstrated a significant difference for IFN-γ responders (P = 0.0003 for IFN; P = 0.2 for TNF-α). Next, we examined the CD8 T-cell response against IDO5 in the PBMC from the donors included in this study (figure 5C) by ELISPOT assay. Indications of a trend between CD4 and CD8 responses against the two IDO peptides was found although not significant (P = 0.21; Mann-Whitney test). Previously, we have described that CMV-specific CD8^+^ T-cell responses were strongly boosted in the presence of IDO-specific T cells and notably, we found IDO-specific CD8^+^ T-cell responses in three healthy donors, which in addition all had strong CMV-specific CD8^+^ T-cells responses. Hence, we examined if there was any correlation between IDOlong responses and T-cell responses against the most immunogenic HLA class I-restricted CMV-epitopes, i.e. the HLA-A2-restricted epitope CMV pp65_495–503_ (NLVPMVATV), the HLA-A11-restricted epitope CMV pp65_16–24_ (GPISGHVLK), the HLA-A1- and HLA-A24-restricted epitope CMV pp65_341–350_ (QYDPVAALFF) as well as the HLA-A1-restricted epitope CMV pp65_363–373_ (YSEHPTFTSQY) (Figure 5D). Hence, we analysed PBMC from the donors included in this study against reactivity against the CMV-derived epitopes in direct ELISPOT assays without any prior stimulation. A Mann-Whitney test illustrated that there was significant difference in T cell reactivity towards CMV epitopes between IDOlong responders and non-responders (P = 0.04).

### Patient Characteristics

**Figure 6:**
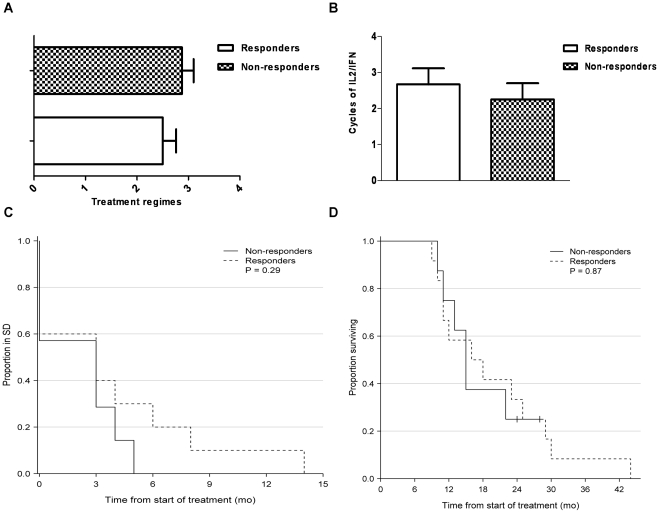
Clinical characteristics of malignant melanoma patients examined for IDOlong responses. *(A),* Previous received number of systemic anti-cancer treatments for the anti-IDO responders and non-responders. Blood samples from melanoma patients were drawn at a minimum of four weeks after termination of any kind of anti-cancer therapy. *(B),* Previous received treatment cycles of IL2/IFN for anti-IDO responders versus non-responders of the malignant melanoma patients. The maximum number of cycles of this therapy is 6 due to severe toxicity. *(C),* Stable disease period (RECIST v.1.0) after treatment with IL2/IFN-α for anti-IDO responders and non-responders. *(D),* Kaplan-Meier survival curve after diagnosis of metastatic disease for anti-IDO responders and non-responders.

From 20 patients with disseminated malignant melanoma we had access to clinical patient records during treatment. Within this group 12 patients had a measurable CD4^+^ T-cell IFN-y ELISPOT response towards IDOlong; whereas 8 patients did not respond. Clinical data are summarized in Table 1. All patients had histological verified stage IV malignant melanoma (UICC TNM Classification) and had measurable disease according to RECIST criteria version 1,0 [20]. In Denmark systemic treatment with high dose Interleukin 2/Interferon (IL-2/IFN – Keilholz decrescendo regime [21]) is 1^st^ line treatment for patients with metastatic malignant melanoma. Other available systemic treatments consist of anti-CTLA-4 antibody (ipilimumab), BRAF inhibitor (vemurafenib), temozolamide and experimental treatments i.e. DC vaccines, CD137 and T-cell therapy. In average, the IDOlong responders had received less overall systemic treatments (2.5 vs. 2.9 regimes) at the time of analysis (Figure 6A). Furthermore, in average patients responding to IDOlong had received more IL-2/IFN treatment than the non-responding patients (3.2 vs. 2.3 cycles), thus indicating a positive correlation between IL-2/IFN treatment and anti-IDO response (Figure 6B). Furthermore, interestingly a trend was found in the IDOlong responding patients towards an extended period of stable disease after IL-2/IFN therapy (SD 3,8 vs. 2,1 months) compared to the non-responding patients, however this did not reach statistical significance (p = 0,28) (Figure 6C). Furthermore, the responders did prove to have a prolonged survival (19.3 vs. 17.3 months) from diagnosis of disseminated malignant melanoma until death, although again not statistically significant (p = 0.87) (Figure 6D).

**Table 1:** Patient characteristics.

Patients No = 20	Responding patients No = 12	Non-Responding Patients No = 8
Age	62 years (49–78)	56 years (38–73)
Gender	F = 3 M = 9	F = 1 M = 7
Stage of disease (IV)	M1a = 0 M1b = 4 M1c = 8	M1a = 0 M1b = 2 M1c = 6
Performance status at leukopheresis	PS 0 = 8 PS 1 = 4	PS 0 = 5 PS 1 = 3
Number of dendritic cell vaccines	Vaccines = 7.9 (5–13)	Vaccines = 8.3 (6–10)
Former treatment with IL2/IFN	Yes = 10	Yes = 7
Number of IL2/IFN	Cycles = 3.2	Cycles = 2.
Response to IL2/IFN	SD = 3.8 months (0–14)	SD = 2.1 months (0–5)
Number of systemic treatments	Regimes = 2.5 (1–4)	Regimes = 2.9 (1–4)
Time from disseminated MM until death	19.8 months (9–44)	17.3 months (10–28)

### IDOlong Contains Various T Cell Epitopes

**Figure 7:**
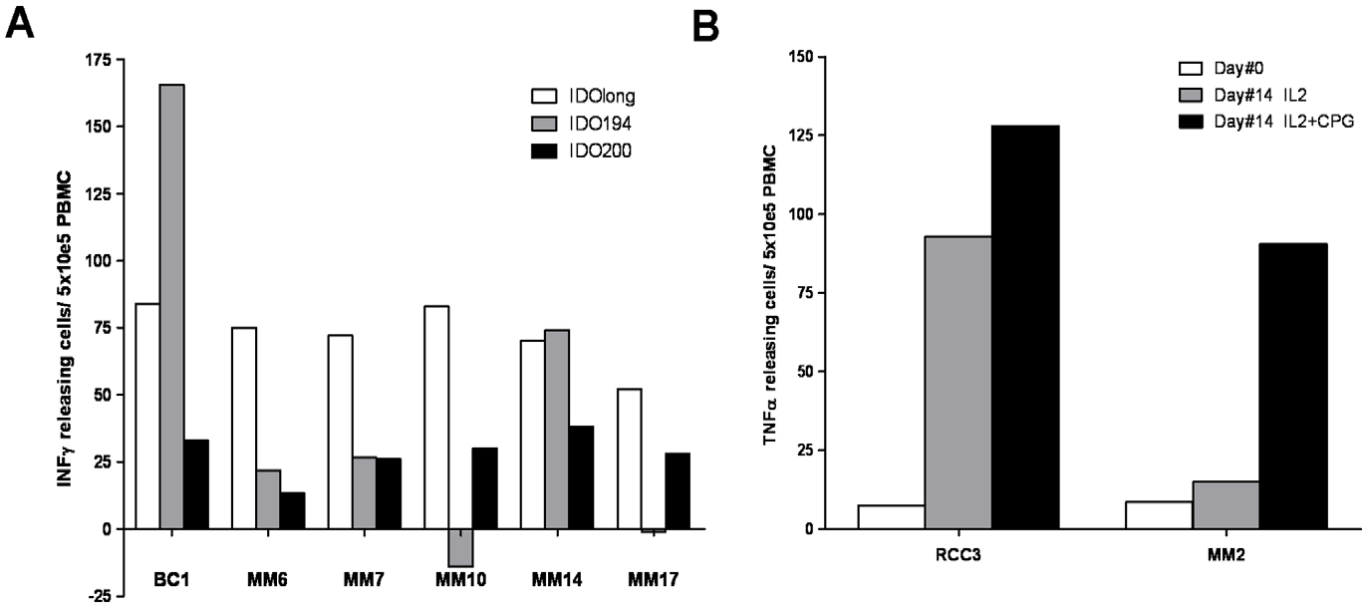
IDOlong contains various T cell epitopes. *(A)* PMBC from seven patients (1 breast cancer patient and 6 melanoma patients) were analysed for responses against IDO194 (DTLLKALLEIASCLE (IDO194-208)) (*grey bars*), IDO200 (LLEIASCLEKALQVF (IDO200-214)) (*white bars*) and compared to IDOlong (*black bars*) by IFN-γ ELISPOT assay. The average number of peptide-specific spots (after subtraction of spots without added peptide) was calculated per 5×10^5^ PBMC for each patient. PBMC were stimulated once with peptide before being plated at 5×10^5^ cells per well in duplicates either without or with the peptide. *(B),* CpG stimulate IDO-specific T cells. PBMC from a melanoma patient and a renal carcinoma patient were treated with either IL-2 or the TLR9 ligand CpG ODN in the presence of IL-2 for 14 days and, subsequently, examined for IDO-specific T cells by TNF-α ELISPOT. Hence, PBMC were plated at 5×10^5^ cells per well in duplicates either without or with the IDOlong peptide before and after cell culture. The average number of TNF-α releasing cells was calculated per 2×10^5^ PBMC for each patient.

The predictive algorithm [19] suggested that two high affinity 15 mer class II epitopes were contained in IDOlong namely DTLLKALLEIASCLE (IDO194-208) and LLEIASCLEKALQVF (IDO200-214). We analysed 7 cancer patients (1 breast cancer patient and 6 melanoma patients) for responses against these 15 mer peptide. In two patients a strong response against DTLLKALLEIASCLE (IDO194-208) were detected whereas weak responses against LLEIASCLEKALQVF (IDO200-214) were detected in most patients (Figure 7A). Interestingly, in most patients IDOlong induced stronger responses than the two 15 mer peptides, which could suggest that the predictive algorithm may underestimate the complex regulation of IDO [22].

### CpG Stimulate IDO-specific T Cells

Triggering of functional IDO requires ligation of B7-1/B7-2 molecules on DC by CTLA4/CD28 expressed on T cells [23]. TLR9 ligation activates DC to up regulate surface expression of B7 ligands and thereby increase expression of IDO [24]. To determine whether this TLR9 ligand-induced up regulation of IDO expression additionally activates CD4 IDO-specific T cells, PBMC from three cancer patients were treated with the TLR9 ligand CpG ODN in the presence of IL-2 for 14 days and, subsequently, examined for IDO-specific T cells. TLR9 signaling with CpG ODN induced a measurable number of IDO-specific T cells in two of the patients (Figure 7B).

### Spontaneous IL-4 or IL-10 Release in Response to IDOlong Peptide

**Figure 8:**
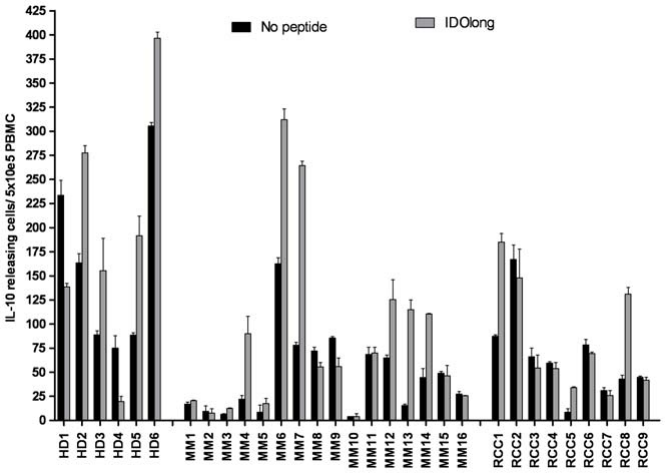
T-cell responses against IDOlong as measured by IL-10 ELISPOT. PBMC from six healthy individuals (HD1-6), sixteen melanoma patients (MM1-16) and nine renal cell carcinoma patients (RCC1-9) were analyzed. PBMC were stimulated once with peptide before being plated at 5×10^5^ cells per well in duplicates either without (*black bars*) or with the IDOlong peptide (*grey bars*).

We examined PMBC from 12 individuals for release of IL-4 in response to IDOlong stimulation. However, we could not detect IL-4 response in any of the examined samples (data not shown). Next, we examined the release of IL-10 in response to IDOlong (Figure 8). We could detect IL-10 release especially in some melanoma patients as well as healthy donors (Figure 8). Notably, in two healthy donors we observed a decrease in IL-10 release compared to background levels.

## Discussion

IDO is an immunoregulatory enzyme that is implicated in immunity under normal and pathological settings [6]. It was recently shown that IDO provides a potential mechanism for the development of DC-mediated T-cell tolerance [25]. IDO^+^ DC inhibit T-cell proliferation due to tryptophan depletion and accumulation of toxic tryptophan metabolites [6]. We have recently described that peptides comprised in the IDO protein sequence are spontaneously recognized by CD8^+^, cytotoxic T cells in cancer patients [15] and healthy individuals [16]. IDO reactive CD8^+^ T cells were able to recognize and kill IDO expressing cells, e.g. tumor cells or regulatory DC. Importantly, the presence of such IDO-specific CD8^+^ T cells boosted T-cell immunity against viral or tumor-associated antigens by eliminating IDO^+^ suppressive cells thereby directly targeting the IDO-dependent counter-regulatory pathway [16]. In the present story, we describe that a long peptide spanning the region around the previously described HLA-A2-restricted epitope IDO199-207 are spontaneously recognized by CD4^+^ T cells both in cancer patients as well as healthy individuals.

We show that IDO-specific CD4^+^ T cells released IFN-γ as well as TNF-α. Hence, the data described here could suggest that such CD4^+^ T cells may play a supporting function by releasing Th1 cytokines in response to a peptide from an immune suppressive molecule like IDO. Although, we were able to detect both IFN-γ and TNF-α response towards IDOlong in healthy donors, the responses were more frequent in cancer patients. It is at this time impossible to determent if the differences in the observed T-cell responses between malignant melanoma and renal cell carcinoma patients may reflect tumor associated effects or individual heterogeneity. In this respect, we describe that IDOlong reacting T-cells in addition react towards DC pulsed with IDO^+^ tumor lysates confirming the relevance of these T cells. We could in contrast not detect any release of the Th2 cytokine IL-4 in response to the IDO-derived peptide. IDO may be critical for the strength and duration of a given immune response due to its inflammation-induced counter-regulatory function. Hence, we speculate that CD4^+^ IDO-specific T cells releasing pro-inflammatory cytokines may play an important role in the early phases of an immune response as a counter-response to the induced regulatory response facilitated by IDO^+^ cells thereby delaying local immune suppression. To this end, the activation of an IDO-specific CD4^+^ Th1-response could prevail over the immune suppressive actions of the IDO-protein, which are otherwise a result of the early expression of IDO in maturing DC or macrophages.

It might be expected that IDO-specific T-cells themselves might be vulnerable for IDO-mediated immune suppression or apoptosis. In this regard, the activation or inhibition of IDO-specific T-cells may depend on a delicate balance; IDO-specific T-cells may become functional when IDO are present in relative small amount but they are inhibited like other T-cells when high amount of IDO and/or other immune suppressive factors (e.g. TGF-β) are present. In should,however, be mentioned that synovial auto reactive T cells have been described in Rheumatoid Arthritis which were resistant to IDO-mediated inhibition [26] due to enhanced storage of intracellular tryptophan in such T-cells. It can be speculated that IDO-specific T-cells are sustained *in vivo* in a similar manner.

We show that the CD4^+^ T cell responses against the IDOlong peptide indeed were HLA class II-restricted and, interestingly, in three donors such IDO-specific, CD4^+^ T cell responses T were detectable in direct *ex vivo* assays. In this regard, it should be noted that with a few exceptions it has not been possible to detect conventional tumor antigen specific T cells in PBMC from cancer patients directly *ex vivo*, i.e. without any *in vitro* steps to expand or enrich these cells [27]. Thus, at least in some donors CD4 positive, IDO-specific T cells were present in relative high frequencies.

Although, it has been described that T-cell epitopes often cluster together at specific immunogenic regions it should be stressed that we have only examined a small region of IDO and, consequently, other IDO-derived CD4 epitopes most likely exist. Hence, the frequency of donors with detectable levels of IDO specific T-cells are most likely higher. The IDOlong peptide contains the HLA-A2-restricted IDO-derived epitope named IDO5 (IDO199-207). We could detect a correlation between patients harbouring IDOlong responses and patients having the HLA class I tissue type HLA-A2. We in addition detected an association between IDOlong and IDO5 responses, which taken together suggest that class I and class II restricted IDO responses co-develop.

We further described frequent IDO-specific CD4^+^ T-cell responses when examining for IL-17 release upon stimulation with IDOlong peptide. IL-17 has been of great interest recently owing to the discovery that the production of IL-17 characterizes a subset of CD4^+^ T-helper cells (Th17 cells). It is well described that Th17 cells contribute to autoimmunity [28]. However, one of the main roles of Th17 cells is to promote host defense against infectious agents, and Th17 cells are thought to be particularly important in maintaining barrier immunity at mucosal surfaces such as in the lungs, gut and skin [29]. Interestingly, IDO is expressed at high levels in the gastrointestinal tract, although its precise role in intestinal immunity is not well understood [30]. The data presented here could suggest that the IDO-specific T cells could be a part of the Th17 cells, which are highly prevalent at the mucosal tissues of healthy individuals [29]. In cancer, Th17 cells might have a protective role in tumor immunopathology by promoting antitumor immunity. Tumor-infiltrating Th17 cells express other cytokines in addition to IL-17, which might be functionally relevant [31], [32]. There was no apparent difference in the number of IL-17 releasing IDOlong reactive T-cells between healthy donors and cancer patients. A large fraction of Th17 cells produce high levels of effector cytokines such as IL-2, IFN-γ as well as TNF [33]. The data presented here suggest that IDO-specific Th17 cells exhibit a similar effector T cell cytokine profile.

IDO is known to be induced by both type I and II interferon’s [34], [35]. Interestingly, it seemed that a higher number of IDO responses were detected among melanoma patients that had received more immune-related treatments (i.e. IL-2, IFNα). Furthermore, the IDO-responding patients seem to have an extended period of stable disease after IL-2/IFNα therapy and an overall prolonged survival although this was not statistical significant. Finally, we were able to directly link the up regulation of IDO with IDO-specific T cells by showing that the addition of the IDO-inducing CpG ODN generated measurable numbers of IDO-specific CD4 T cells among PBMC *in vitro*.

The CD8^+^ T-cell response to CMV typically constitutes a sizeable percentage of the CD8^+^ T-cell repertoire in CMV-seropositive individuals [36]. CMV has been shown to induce IDO expression in monocytes, which might present an advantage to CMV-infected monocytes to escape T-cell responses [37]. Here we show that the presence of IDOlong responses correspond to the presence of CMV-responses, which could suggest that the IDO specific T cells function as supporter T cells for the constitutive anti-CMV CD8^+^ T-cell response.

Interestingly, in some donors we detected background IL-10 release in *in vitro* pre-stimulated ELISPOT assays. This enabled us to perceive that stimulation with the IDO-derived peptide in two healthy donors triggered an overall suppression of IL-10. In this regard, we have previously observed a decrease in IL-10 when IDO-specific CD8^+^ T cells were present [16]. However, we could in additional donors detect IL-10 release in response to IDOlong peptide. IL-10 is mainly expressed by Tregs that have been defined as a specialized subpopulation of T cells that act to suppress activation of the immune system and thereby maintain immune system homeostasis and tolerance to self-antigens. Consequently, it can be speculated that IDO-specific Tregs may enhance the IDO-mediated immune suppression protecting cells from an immune attack. Hence, the role of IDO-specific CD4^+^ T cells in immune-regulatory networks may be a complex balance between activation and inhibition depending on the microenvironment. In this respect, it was recently described that IDO play biologically important roles in the host response to diverse intracellular infections but in addition that the nature of this role – antimicrobial or immune regulatory - might depend on the pathogen. Hence, IDO inhibition might not always benefit the host. In this regard, IDO inhibition during murine toxoplasmosis led to increased mortality with increased parasite burdens [38]. This should naturally be taken into account when exploring the possible use of IDO-specific T-cells in the clinic.

In conclusion, we describe the presence of naturally occurring IDO-specific CD4^+^ T-helper cells in cancer patients and in healthy donors. We suggest that the activation of such CD4^+^ cells could help overcoming the immune suppressive actions of the IDO-protein, which are otherwise a result of the early expression of IDO in maturing antigen-presenting cells. However, since IDO-specific CD4^+^ T cells in addition may secrete regulatory cytokines the balance between activation and inhibition may vary depending on the phenotype that yet again may depend on the microenvironment.
